# Cholecystokinin Modulates Corticostriatal Transmission and Plasticity in Rodents

**DOI:** 10.1523/ENEURO.0251-24.2025

**Published:** 2025-03-06

**Authors:** Chloé Guillaume, María Sáez, Patricia Parnet, Ramón Reig, Vincent Paillé

**Affiliations:** ^1^Nantes Université, INRAe, UMR 1280 PhAN, IMAD, Nantes F-44000, France; ^2^Instituto de Neurociencias UMH-CSIC, San Juan de Alicante 03550, Spain

**Keywords:** basal ganglia, cholecystokinin (CCK), corticostriatal synapse, spike-timing–dependent plasticity

## Abstract

Recent findings have shifted the view of cholecystokinin (CCK) from being a cellular neuronal marker to being recognized as a crucial neuropeptide pivotal in synaptic plasticity and memory processes. Despite its now appreciated importance in various brain regions and abundance in the basal ganglia, its role in the striatum, which is vital for motor control, remains unclear. This study sought to fill this gap by performing a comprehensive investigation of the role of CCK in modulating striatal medium spiny neuron (MSN) membrane properties, as well as the secondary somatosensory cortex S2 to MSN synaptic transmission and plasticity in rodents. Using in vivo optopatch-clamp recording in mice on identified MSNs, we showed that the application of CCK receptor Type 2 (CCK2R) antagonists decreases corticostriatal transmission in both direct and indirect pathway MSNs. Moving to an ex vivo rat preparation to maximize experimental access, we showed that CCK2R inhibition impacts MSN membrane properties by reducing spike threshold and rheobase, suggesting an excitability increase. Moreover, CCK modulates corticostriatal transmission mainly via CCK2R, and CCK2R blockage shifted spike-timing–dependent plasticity from long-term potentiation to long-term depression. Our study advances the understanding of CCK's importance in modulating corticostriatal transmission. By showing how CCK2R blockade influences synaptic function and plasticity, we provide new insights into the mechanisms underlying striatal functions, opening new paths for exploring its potential relevance to neurological disorders involving basal ganglia-related behaviors.

## Significance Statement

Cholecystokinin (CCK) plays a critical role in synaptic plasticity and memory but completely unexplored in corticostriatal synapses and motor control. This study shows that blocking the CCK receptor Type 2 reduces excitatory postsynaptic potentials and excitatory postsynaptic currents in the motor striatum (in vivo and ex vivo) and disrupts corticostriatal spike-timing–dependent plasticity, shifting it from long-term potentiation to long-term depression. These findings reveal CCK signaling as a key modulator of corticostriatal communication, capable of reversing the direction of synaptic plasticity. The results position CCK as a crucial regulator of synaptic and motor functions, with implications for understanding corticostriatal mechanisms.

## Introduction

Over 100 neuropeptides have been identified in the human brain, representing one of the largest and most diverse classes of signaling molecules (Merighi et al., 2011; Russo, 2017). While numerous studies independently demonstrated neuropeptide involvement at both the cellular and behavioral levels (van den Pol, 2012; Bucher and Marder, 2013; Nadim and Bucher, 2014; Rilling and Young, 2014; Muir et al., 2024), their pivotal neuromodulatory role is often overlooked. This is likely due to the experimental challenge of addressing the complexity and diversity of the action mechanisms, as well as their varied expression profiles (van den Pol, 2012; Bucher and Marder, 2013; Muir et al., 2024). To reduce this complexity to an experimentally tractable level, we chose to focus this study on the most abundant neuropeptide in the brain, cholecystokinin (CCK; Beinfeld et al., 1981; Moran and Schwartz, 1994).

CCK is widely distributed across the brain, and it is especially concentrated in the striatum, cortex, and limbic areas (Vanderhaeghen et al., 1980; Beinfeld et al., 1981; Crawley, 1985). Previous studies have reported CCK's involvement in synaptic transmission and plasticity modulation in the cortex (Siniscalchi et al., 2003; Gallopin et al., 2006; Chung et al., 2009; Chen et al., 2019), hippocampus (Balschun and Reymann, 1994; Migaud et al., 1994; Yasui and Kawasaki, 1995; Breukel et al., 1997; Deng and Lei, 2006; Deng et al., 2010), ventral tegmental area (Martinez Damonte et al., 2023), and hypothalamus (Crosby et al., 2018). Surprisingly, given its predominance there, little is known about its role in striatum, a key hub for motor control and cognitive functions (Calabresi et al., 1996; Kreitzer and Malenka, 2008; Di Filippo et al., 2009; Reig and Silberberg, 2016). It is also rich in a specific CCK receptor, CCK receptor Type 2 (CCK2R; Van Dijk et al., 1984; Pélaprat et al., 1987).

As the primary input structure of the basal ganglia, the striatum receives substantial projections from different cortical areas, prompting us to focus our work on the corticostriatal synapse, specifically between secondary somatosensory cortex (S2) and striatal medium spiny neuron (MSN). This is a crucial cortical–subcortical synaptic hub whose dysfunction has been reported in various disorders such as Huntington's (Laforet et al., 2001; Miller et al., 2011; Hong et al., 2012) and Parkinson's (Day et al., 2006; Azdad et al., 2009; Suarez et al., 2016; Villalba and Smith, 2018), among others. Numerous molecular players have been shown to be involved in the function of this S2→MSN synapse and its plasticity (Fino and Venance, 2010; Fino et al., 2010; Paille et al., 2013; Cui et al., 2015), particularly glutamate, dopamine, and acetylcholine but also nitric oxide and endocannabinoids. This suggests that the corticostriatal S2→MSN synapse is highly modulated, likely also by CCK.

To address this gap in the literature and understand how the brain's most abundant neuropeptide acts on corticostriatal communication, we investigated the potential role of CCK and its receptor CCK2R in corticostriatal synaptic transmission and plasticity in ex vivo and in vivo rodent models. Overall, our results showed that CCK strongly influences MSN membrane properties as well as S2→MSN corticostriatal transmission and plasticity. Indeed, we observed that blocking CCK2R impacts MSN excitability and shortens S2→MSN excitatory postsynaptic currents (EPSCs). Moreover, it reverses spike-timing–dependent plasticity (STDP) direction from long-term potentiation (LTP) to long-term depression (LTD).

## Materials and Methods

### Animals

Sprague Dawley rats of both sexes between the ages of 14 and 35 d were used for ex vivo electrophysiology experiments. Transgenic mice D2-cre × ChR2 of both sexes were used between 2 and 4 months of age to induce the expression of channelrhodopsin (ChR2) for the identification of direct and indirect MSNs, as previously shown (Ketzef et al., 2017; Alegre-Cortés et al., 2021). No difference between males and females was observed in any of the analyzed parameters (all *p* > 0.3). All experimental procedures were conducted in accordance with the European Guidelines and Directive 2010/63/EU, as well as the RD 53/2013 Spanish regulation, evaluated by the local ethical committee on animal experimentation (CEEA PDL) and approved by the French Ministry of Superior Education and Research (APAFIS #30245-202104291141254v1).

### Pharmacology

LY225910 (LY; Tocris Bioscience), a potent CCK2R antagonist (IC_50 _= 9.3 nM), was first dissolved in DMSO as a stock solution and then used at the following working dilution based on previously published work: 1 µM in ACSF for ex vivo electrophysiology experiments (Crosby et al., 2018) and 0.1 mg/kg administered intraperitoneally for in vivo electrophysiology experiments (Farook et al., 2004). Lorglumide (Lorg; Sigma-Aldrich), a CCK1R antagonist, was added to the extracellular solution to obtain the perfusion solution of 1 µM during patch-clamp recording ex vivo (Crosby et al., 2018).

### In vivo electrophysiology

Mice were anesthetized by an intraperitoneal injection of ketamine (75 mg/kg) and medetomidine (1 mg/kg) diluted in 0.9% NaCl. A maintaining dose of ketamine (30 mg/kg, i.p.) was administered every 2 h or after changes in the electroencephalogram or reflex responses to paw pinches. Mice were placed in a stereotaxic frame (Stoelting), and air enriched with oxygen was delivered through a thin tube. The temperature was maintained at 36.5 ± 0.5°C using a feedback-controlled heating pad (FHC). Three craniotomies were drilled at the following coordinates from the bregma: AP 0 mm and LM 4 mm for dorsolateral striatum (DLS), whole-cell recordings; AP −0.7 mm and LM 3.8 mm for ipsilateral S2, stimulation; and AP −0.7 mm and LM −3.8 mm for controlateral S2, local field potential (LFP) recordings (Paxinos and Franklin, 2001). The exposed brain was continuously covered by 0.9% NaCl to prevent drying. Whole-cell patch–clamp recordings were obtained from the DLS between 2.068 and 2.251 µm depth, with an electrode angle of ∼30°. Signals were amplified using a MultiClamp 700B amplifier and digitized at 20 KHz with a Cambridge Electronic Design acquisition board and Spike2 software. Borosilicate pipettes (1B150F-4, World Precision Instruments) had a resistance of 6–12 MΩ when backfilled with intracellular solution containing the following (in mM): 125 K-gluconate, 10 KCl, 10 Na-phosphocreatine, 10 HEPES, 4 ATP-Mg, and 0.3 GTP-Na. pH and osmolarity were adjusted to 7.4 and 280 mOsm/L, respectively. In addition, biocytin (0.2–0.4% Sigma-Aldrich) was diluted for post hoc staining. The average recording time for all MSNs was 68.6 ± 34.95 min (minimum, 42 min; maximum, 156 min; *n* = 23). Recorded MSNs were optogenetically identified and classified as a putative direct or indirect MSN by means of the optopatcher (Katz et al., 2013). One or two serial pulses with five light steps of 500 ms each were delivered every 2 s with increasing intensity from 20 to 100% of full LED power (SLA-1000-2, FCS-0470-000, Mightex Systems; minimal light power of 0.166 mW; maximal power of 0.83 mW at the tip of the fiber) measured with a power meter console (PM100D, Thorlabs). Once the neuron identity was confirmed, their electrophysiological properties were investigated by injecting positive and negative intracellular current steps. Their synaptic connectivity was then assessed via electrical stimulation of S2 with a bipolar electrode. The stimulation consisted of a pulse of 10 ms duration and 6.5 µA intensity. The analysis of any effect on the spontaneous activities or in the peri stimulus time histogram evoked potential was conducted at least 15 min postinjection. At the end of the experiment, animals were killed and processed for immunohistochemistry.

### Immunohistochemistry and tracing

Mice were killed with a lethal dose of sodium pentobarbital (200 mg/kg, i.p.) and perfused with a solution containing 4% paraformaldehyde in 0.1 M phosphate buffer (PB), pH 7.4. Brains were extracted and stored in a PB saline (PBS) solution then in PBS containing 30% sucrose for 48 h. Coronal slices (20 µm thickness) containing the entire striatum from the recorded side (from AP 1.4 mm to AP −1.3 mm, following Paxinos and Franklin, 2001), were obtained using a digital automatic cryotome (Leica) and collected on gelatin-coated glass slides. Sections were incubated overnight with Cy3-conjugated streptavidin diluted (1:1,000) in 1% bovine serum albumin (BSA) and 0.03% Triton X-100 in 0.1 M PBS mounted and imaged. Neurons were then reconstructed using the widefield THUNDER Imager microscope (Leica Microsystems), and the images were processed using the ImageJ software.

To trace axons, four C57BL/6J mice were anesthetized with isoflurane while immobilized in a stereotaxic frame. A 300 nl of biotinylated dextran amines (BDA, 10%, Sigma-Aldrich) was injected unilaterally in S2 at the following coordinates: AP −0.7 mm, LM 3.8 mm, and DV −0.8 mm. Ten days after the injection, they were killed and processed as described above to obtain coronal slices (50 µm thick) containing both hemispheres. Sections were incubated overnight with Cy3-conjugated streptavidin diluted (Jackson ImmunoResearch Laboratories, 1:1,000) in 1% BSA and 0.03% Triton X-100 in 0.1 M PBS, mounted and imaged with the widefield THUNDER Imager microscope (Leica Microsystems) and the Leica Application Suite X software.

### Ex vivo electrophysiology

Animals were anesthetized with an intraperitoneal injection of 0.1 ml of pentobarbital and then decapitated 5 min later. The brain was rapidly extracted, placed in a frozen oxygenated ACSF (in mM: 125 NaCl, 2.5 KCl, 2 CaCl_2_, 1 MgCl_2_, 5 NaHCO_3_, 25 glucose, 1.25 NaH_2_PO_4_), and sectioned into 300 µm horizontal brain slices with a Leica VT1000S Vibratome to preserve intact connections between the cortex and striatum (Fino et al., 2005; Paille et al., 2013). Slices were placed in an oxygenated ACSF solution at 32°C. In the electrophysiology station, slices were continuously perfused with ACSF solution. Using a micromanipulator (Sutter Instrument), a borosilicate glass pipette (GC150TF-10, Harvard Apparatus) filled with the intracellular solution (in mM: 122 K-gluconate, 13 KCl, 10 HEPES, 4 MgATP, 0.3 NaGTP, 10 phosphocreatine), pH 7.36, was placed on the MSN imaged through an Olympus BX51 microscope with a 40× water-immersion objective. Patch-clamp recording steps were made as previously described using an EPC10-USB HEKA amplifier and acquisition software PatchMaster (Venance et al., 2004; Fino et al., 2005, 2008). Recordings were corrected for a junction potential of +18 mV. The series resistance was monitored throughout the experiment by a brief voltage step of −5 mV at the end of each recording. MSNs were recorded in the dorsolateral region of the striatum and identified thanks to their specific spiking pattern and electrophysiological properties: (1) an hyperpolarized resting membrane potential (76.95 ± 1.37 mV), (2) an inward rectification, as depicted in the steady-state current–voltage (*I*–*V*) relationship, (3) a prolonged depolarizing ramp toward the AP threshold that gives rise to a delayed onset of the first spike.

For electrical stimulation, a bipolar electrode (CBDSF7, FHC) was placed in Layer V of the secondary somatosensory cortex (S2). Cortical stimulations were monophasic and at constant current between 3 and 32 mA with a duration of 100–150 µs. After the 20 min recording of a stable MSN EPSCs (baseline), LY or Lorg was added into the bath (baseline + drug). After the recording of stable EPSCs (baseline amplitude was set at ∼150 pA), a STDP protocol was applied by pairing pre- and postsynaptic stimulations repeated 100 times at 1 Hz with a time shift of a few milliseconds (Δ*t*). Then, EPSCs were recorded for 60 min. EPSC amplitudes following plasticity induction were compared with baseline amplitudes recorded in the presence of the drug to evaluate the occurrence of long-term synaptic plasticity. Throughout the protocol, input resistance (Ri) was continuously monitored, with experiments excluded if Ri varied by ∼20%. Signals were digitized at 10 kHz. Data analysis of the recordings was performed with custom-written scripts in MATLAB.

### Statistical analysis

All statistical analyses were conducted with GraphPad Prism 9, data are presented as mean ± SEM, “*n*” symbolizes the number of cells, and statistical significance was defined as *p* < 0.05. Statistical evaluation of the effect of the drug on EPSC was performed by comparing EPSCs before drug infusion and after drug infusion using Wilcoxon signed rank. Statistical evaluation of the occurrence of long-term plasticity was performed on the baseline measurement after drug addition and before STDP, at the latest moment where the cell is still alive (5 min selected from 30 min to 60 min after STDP) using *t* test (compare with 100% baseline) and Mann–Whitney test to compare groups. To define the locus of synaptic plasticity, CV^2^ was calculated as described by Brock et al. (2020): CV^2 ^= (SD / µ)^2^, where *µ* is the mean. For the in vivo electrophysiology, statistical evaluation of the effect of the drug on excitatory postsynaptic potential (PSP) was performed on baseline measurements before injection and 15 min after drug injection using the Wilcoxon signed rank test.

## Results

### In vivo whole-cell patch–clamp recording of identified MSNs in the DLS

**Figure 1.**
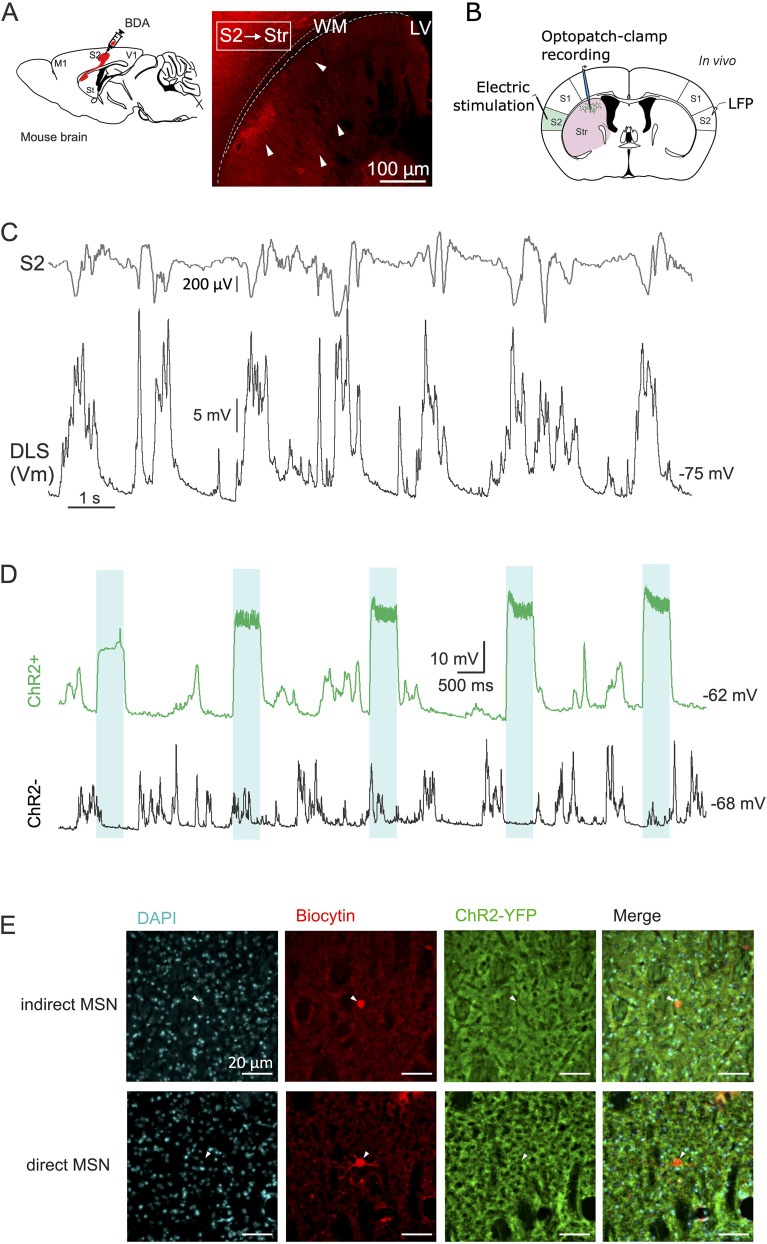
In vivo classification of direct and indirect pathway murine MSNs and confirmation of S2 projections to MSNs. ***A***, Confirmation of substantial S2 projections to the DLS via BDA (red) injection in S2, showing axonal boutons present in the striatum (showed with white arrows). Str, striatum; WM, white matter; LV, lateral ventricle. ***B***, Schematic representation of the stimulation electrode placement in S2, optopatch pipette in dorsal striatum, and LFP electrode in the contralateral S2. In total 38 MSNs (21 D1 MSNs and 17 D2 MSNs) have been recorded. ***C***, Representatives of LFP traces in S2 and of whole-cell optopatch–clamp traces in the striatum. ***D***, Classification of D2 (ChR2+, top) and D1 (ChR2−, bottom) MSNs based on the presence or absence of membrane depolarization in response to pulsed blue light (vertical shading). ***E***, Postmortem validation of D1 (ChR2−) or D2 (ChR2+) MSN identity through immunostaining against biocytin with streptavidin-Cy3 (red) and DAPI (blue) to unveil the recorded neuron and the neuronal nuclei, respectively. Notice how D2 ChR2+ cells display fluorescence for YFP (green), showing its positiveness for ChR2.

Although significant amounts of CCK and CCK2R are expressed in the cortex and striatum, the role of CCK in modulating the corticostriatal synapse—a crucial hub for behavioral output—remains unknown. We first confirmed with BDA axonal tracing that S2 neurons project monosynaptically to MSNs in the striatum (Fig. 1*A*) and localized the striatal region in which the axonal density was highest. We thus asked if the CCK2R inhibition was able to disrupt this S2→MSN synapse transmission and/or if CCK modulation had differential effects on S2 inputs onto direct and indirect MSN striatal neuronal types (Hersch et al., 1995; Surmeier et al., 1996; Valjent et al., 2009; Bertran-Gonzalez et al., 2010). The direct and indirect pathways target different nuclei within the basal ganglia, influencing movement initiation and inhibition (Albin et al., 1989). Notably, they express different complements of dopaminergic receptors, making the differential expression of CCK2R a plausible possibility. Thus, to classify direct and indirect MSNs during in vivo electrophysiological recordings, we combined in vivo patch-clamp recording of MSNs with optogenetic stimulation via a fiber inserted inside the recording electrode (optopatch) in D2-ChR2 adult mice which were also equipped with a bipolar electrode in S2 for cortical stimulation (Fig. 1*B*). Cells in the indirect pathway express D2 receptors, so were ChR2 positive, and responded to blue light stimulation by depolarizing their membrane potential. Conversely, cells in the direct pathway express D1 and were ChR2-negative and so were nonresponsive to blue light stimulation (Fig. 1*D*). The identity of D1 and D2 MSNs was confirmed through immunohistofluorescence, combining ChR2 (green, i.e., D2 receptor-positive neurons) immunostaining with the visualization of the patched neuron filled with biocytin (Fig. 1*E*). We obtained in vivo whole-cell patch–clamp recordings from both types MSNs located in the DLS. All of them displayed slow-wave oscillations with prominent Up and Down states (Fig. 1*C*,*D*) at a frequency of 0.93 Hz (with no significant differences between D1 and D2 MSNs; 0.97 Hz and 0.83 Hz, respectively; *p* = 0.65). The whole group presented an average input resistance of 404 ± 24.60 MΩ with a significant higher average resistance for D1 MSNs (471 ± 31.13) compared with D2 MSNs (321 ± 69.42); *p* = 0.0026. The whole group presented an average capacitance of 30.54 ± 15.76 pF and an average Tau of 8.87 ± 2.44 ms without significant difference by subgroups.

### CCK2R blockage differentially modulates S2→MSN transmission in the direct and indirect pathway

**Figure 2.**
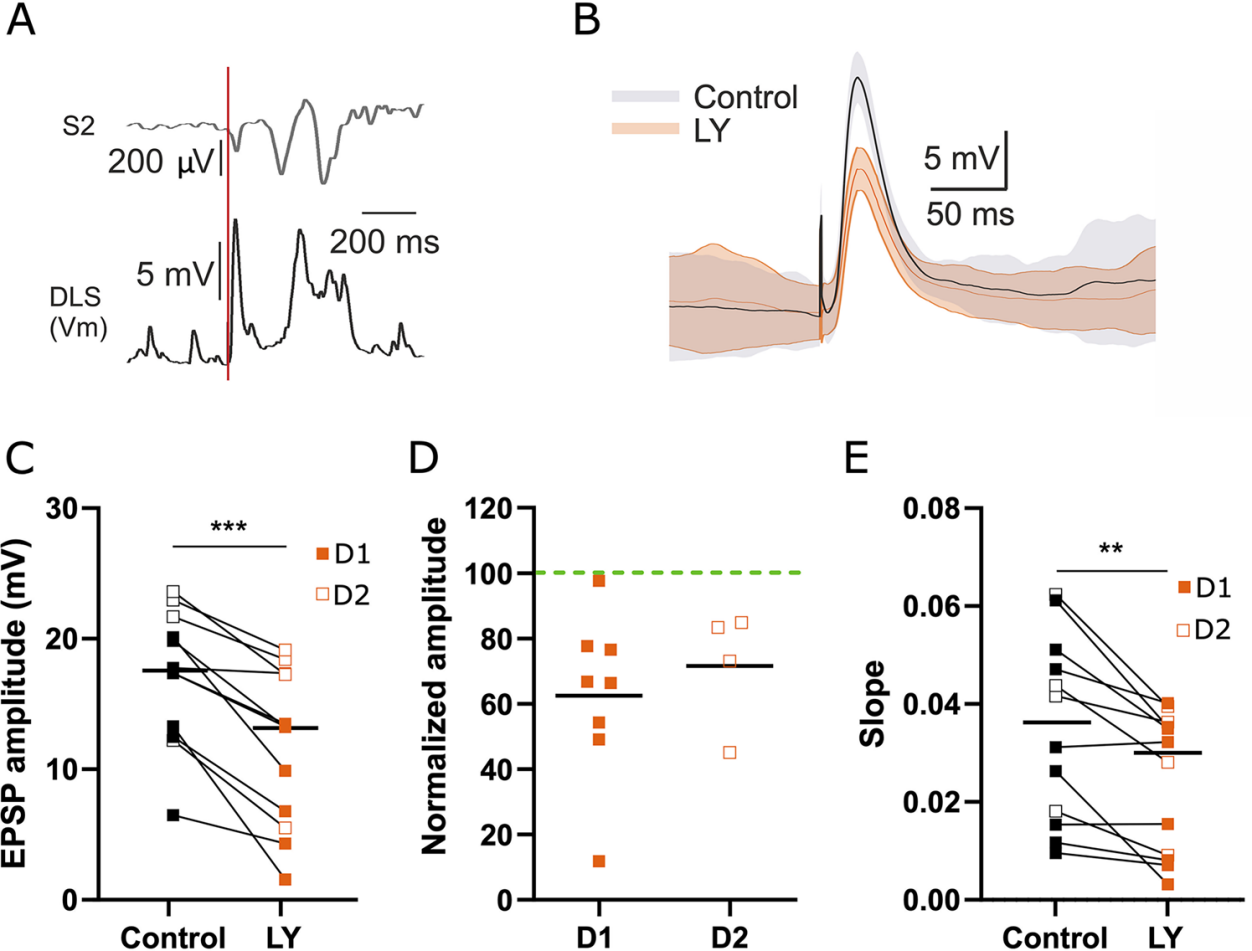
LY IP injection reduces evoked PSP amplitude in direct and indirect MSNs in vivo. ***A***, Representative trace of whole-cell recordings in DLS and of contralateral S2 LFP following an electrical stimulation in ipsilateral S2 (red line). ***B***, Average (thick line) and variance (shading) MSN PSPs in downstate before and after injection of CCK2R antagonist LY (orange line). ***C***, Effect of LY injection on MSN PSPs amplitude in cells from both direct (D1) and indirect (D2) MSNs (*n* = 12). ***D***, On normalized PSP amplitude in direct (D1, *n* = 8) and indirect (D2) MSNs (*n* = 4). Significant diminution of the PSP slope in both direct (D1) or indirect (D2, *n* = 4) MSNs and on ***E***, PSP slope in combined D1 and D2 MSNs (*n* = 12). Dots and squares are individual cells before (black) and after (orange) LY application; **p < 0.01; ***p < 0.001.

We proceeded to investigate the effect of CCK on S2→MSN synaptic transmission in vivo. To achieve this, we electrically stimulated S2 and recorded the identified MSNs using the optopatcher. We first acquired baseline recordings of PSPs evoked in the dorsal striatum by S2 electrical stimulation (pulse of 10 ms duration and 6.5 µA intensity); then we injected the selective CCK2R antagonist LY225910 (LY) intraperitoneal, at 0.1 mg/kg concentrations previously described and validated in the literature (Farook et al., 2004; Fig. 2*A*,*B*). LY administration resulted in a significant reduction in PSP amplitude (Fig. 2*B*,*C*; control = 17.10 ± 1.48 mV, LY = 11.69 ± 1.73 mV; *p* = 0.0005), together with a significant diminution of the slope (Fig. 2*E*; control = 0.035 ± 0.005; LY = 0.024 ± 0.004; *p* = 0.0024). Interestingly, the amplitude decrease occurred in both D1 and D2 MSNs with no significant difference between groups (D1 MSNs = 62.54% ± 8.98; D2 MSNs = 71.61% ± 9.21; *p* = 0.54; Fig. 2*D*). Moreover, when checking the effect of LY for both sexes, we could not observe any significant effect when comparing male and female recorded cells with their respective control groups (*p* = 0.8, data not shown). These results indicate that in vivo, LY intraperitoneal injection decreases corticostriatal transmission in both direct and indirect pathway MSN populations, without any specific differences regarding the sex.

### CCK2R inhibition impacts MSN membrane-active properties by reducing spike threshold and rheobase

**Figure 3.**
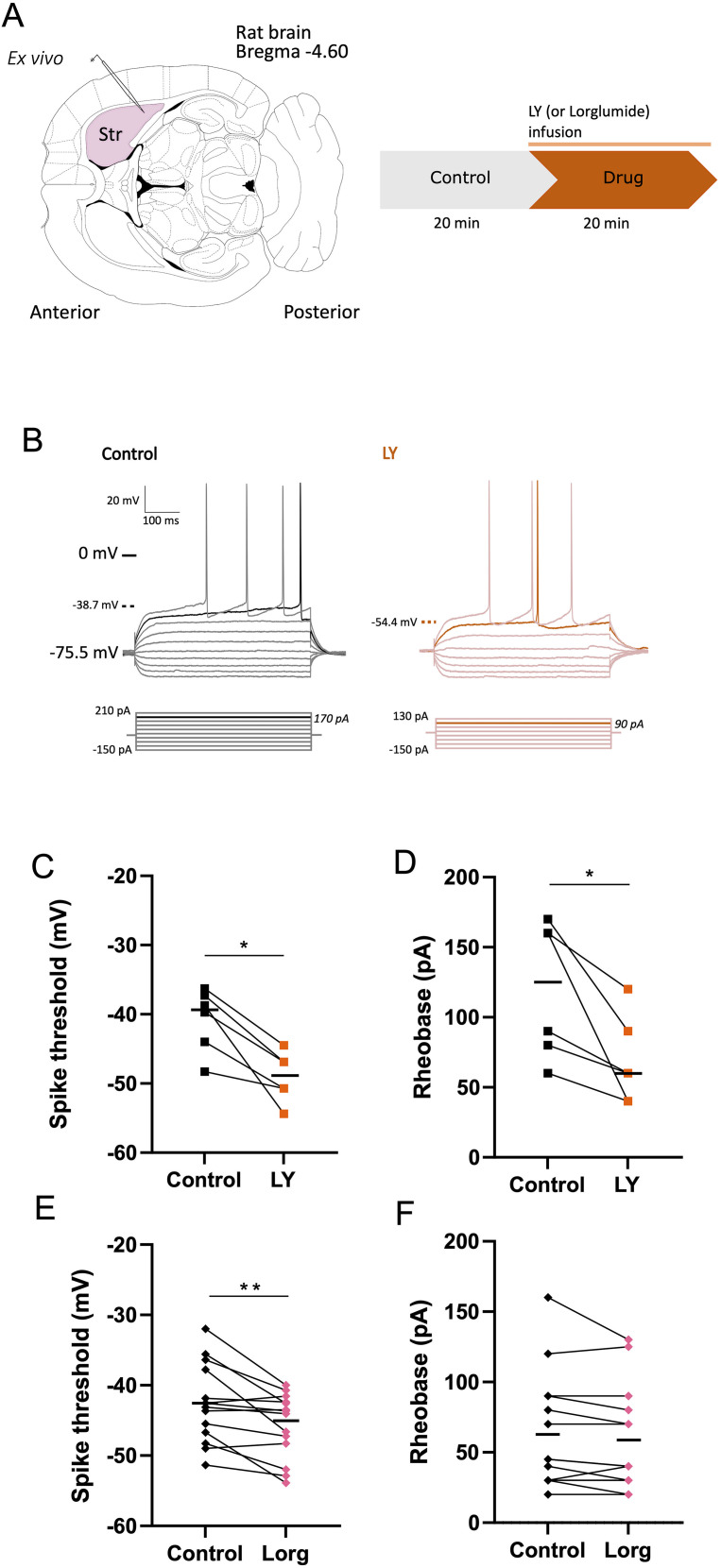
CCK2R antagonist LY application modulates MSN membrane properties ex vivo. ***A***, Left, Schematic representation of the horizontal corticostriatal rat brain slice preparation with the recording electrode in the striatum (Str). Right, Schematic representation of the experience timeline. ***B***, Effect of LY application on MSN spike and rheobase (*n* = 6). ***B***, Example traces of MSN responses to current injections of increasing amplitude before and after LY application. ***C***,***D***, Effect of CCK2R antagonist LY application on MSN spike threshold and rheobase (*n* = 5). Dots and squares are individual cells before (black) and after LY (orange). ***E***,***F***, Effect of CCK1R antagonist Lorg application on MSNs spike threshold and rheobase (*n* = 14). Dots and squares are individual cells before (black) and after Lorg (pink) application; *p < 0.05.

Given the absence of differential CCK modulation between the indirect and direct pathways in vivo, we transitioned to an ex vivo acute slice preparation in rats. This well-described approach enabled a more detailed investigation of the electrophysiological properties of MSNs. While it does not permit MSN subtyping—unnecessary based on our earlier findings—it provides significantly more controlled conditions for electrophysiological recordings and pharmacological studies. MSNs were recorded in the dorsolateral region of the striatum (Fig. 3*A*) before and after bath application of 1 µM of LY (Crosby et al., 2018; Fig. 3*B*). CCK2R antagonist did not significantly affect any passive membrane properties (for before vs after LY infusion, Ri = 253.06 ± 52.76 MΩ vs 220.21 ± 37.29 MΩ; conductance = −0.33 ± 0.28 S vs −0.16 ± 0.16 S; membrane potential = −76.95 ± 1.37 mV vs −76 ± 1.70 mV) but caused a reduction in both spike threshold (control = −40.72 ± 1.87 mV; LY = −49.02 ± 1.46 mV; *p* = 0.0313; Fig. 3*C*) and rheobase (control = 120 ± 19.83 pA; LY = 68.33 ± 12.76 pA; *p* = 0.0313; Fig. 3*D*). Interestingly, these observations were also independent of the sex and were specific for the CCK2R pathway. Indeed, bath application of CCK1R antagonist Lorg (Crosby et al., 2018) did not significantly affect any passive membrane properties (before vs after Lorg infusion, Ri = 353.1 ± 29.22 MΩ vs 375.5 ± 38.98 MΩ; membrane potential = −69.73 ± 1.83 mV vs −69.64 ± 1.76 mV) but caused a small decrease in spike threshold (control = −43.42 ± 1.62 mV; Lorg = −45.65 ± 1.26 mV; *p* = 0.0322; *n* = 14; Fig. 3*E*) while not changing the rheobase (control = 54.55 ± 9.45 pA; Lorg = 53.18 ± 9.68; *n* = 14; Fig. 3*F*).

### CCK modulates corticostriatal transmission mainly via Type 2 receptors

**Figure 4.**
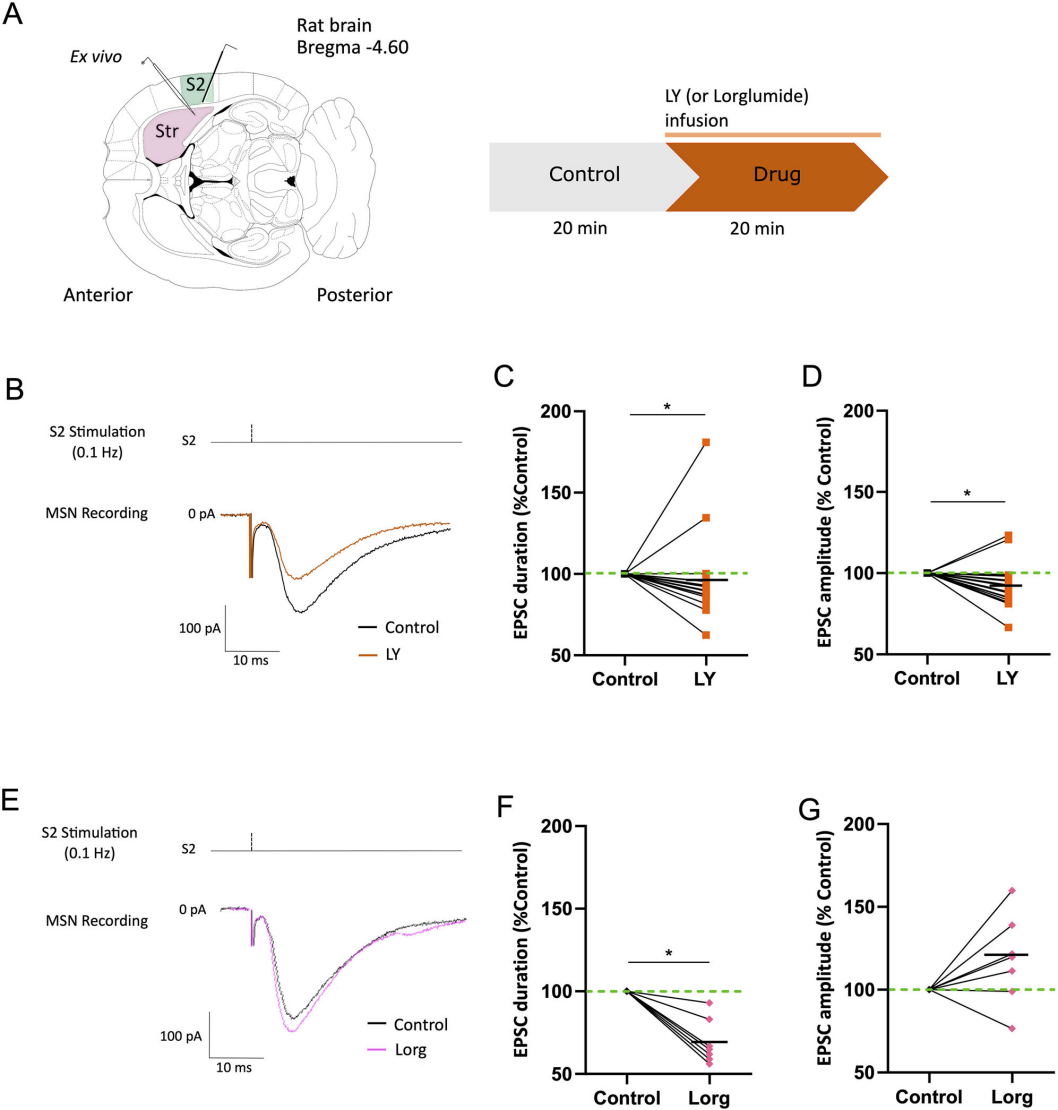
Ex vivo LY application decreases glutamatergic transmission and alters EPSC properties at the S2→MSN synapse. ***A***, Left, Schematic representation of the horizontal rat corticostriatal brain slice preparation with the recording electrode in the striatum and the stimulation electrode placed in S2. Right, Schematic representation of the experience timeline. ***B***, Stimulation protocol (repeated every 10 s, i.e., 0.1 Hz) and evoked glutamatergic response from a representative recorded MSN before (control) and after (LY) LY infusion. ***C***,***D***, Effect of LY application on S2→MSNs EPSC duration and amplitude (*n* = 18). ***E***, Stimulation protocol and evoked glutamatergic response from a representative recorded MSN before (control) and after Lorg application. ***F***,***G***, Effect of Lorg application on S2→MSN EPSC duration and amplitude (*n* = 7). Dots and squares are individual cells before (black) and after LY (orange) or Lorg (pink) application; *p < 0.05.

To confirm that the corticostriatal transmission was affected by CCK, as seen in in vivo patch-clamp observations, we placed a bipolar stimulation electrode in the Layer V of S2 and recorded MSN synaptic response to a single pulse (Fig. 4*A*,*B*; Fino et al., 2005, 2010; Paille et al., 2013). The EPSC duration (from the start of depolarization to the return to prestimulation potential) significantly decreased after CCK2R antagonist LY bath application (control = 45.13 ± 5.77 ms; LY = 42.82 ± 5.53 ms; *p* = 0.0237; Fig. 4*C*). Moreover, the LY application decreased EPSC amplitude in most of them (mean absolute values, control = 166.02 ± 16.57 mV; LY = 153.40 ± 15.71 mV; *p* = 0.0120; normalized mean, control = 100%; LY = 92.98%; *p* = 0.0208; Fig. 4*D*), suggesting that LY affect the corticostriatal transmission, aligned with our in vivo results. To verify the specificity of CCK receptors in modulating synaptic transmission, in a subset of cells, we applied the CCK1R antagonist Lorg (Fig. 4*E*). Blocking CCK1R shortened the EPSC duration (control = 43 ± 5.28 ms; Lorg = 29.61 ± 3.84 ms; *p* = 0.0156; Fig. 4*F*) but did not affect the EPSC amplitude (control = 116.46 ± 14.05 mV; Lorg = 134.39 ± 17.24 mV; Fig. 4*G*).

Taken together, these results indicate that CCK modulates corticostriatal efficiency and modifies MSN excitability, with a major role played by CCK2R which impact on both postsynaptic events and active membrane properties.

### CCK2R inhibition induces a switch on STDP LTP

**Figure 5.**
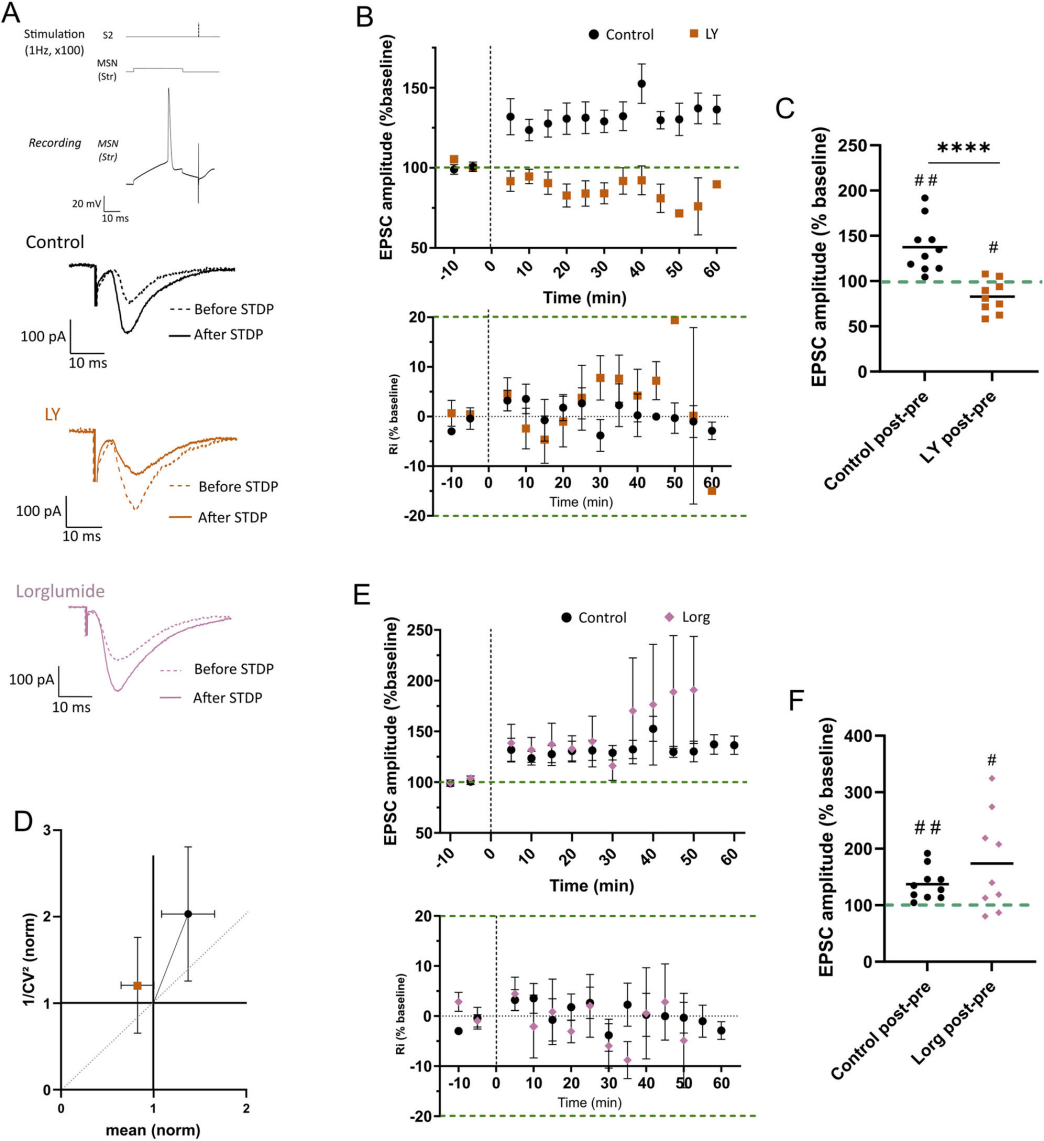
CCK2R blocker LY switches long-term STDP from potentiation to depression. ***A***, STDP protocol, with post–pre pairing repeated 100 times at 1 Hz (top), and evoked EPSCs from a representative MSN during baseline and long-term conditions under control, LY, or Lorg application. ***B***,***C***, Normalized EPSC amplitude (top and right) and input resistance (bottom) to preplasticity induction in control and LY conditions (black control *n* = 10; orange LY *n* = 9). ***D***, Normalized 1/CV^2^ of control condition and LY condition. ***E***,***F***, Normalized EPSC amplitude (top and right) and input resistance (bottom) to preplasticity induction in control and Lorg conditions (black control *n* = 10; pink Lorg *n* = 9). Dots/squares are mean, and error bars are ±SEM; ****p < 0.0001.

Given the impact of a CCK2R inhibition on the corticostriatal transmission as well as the previous reports on CCK involvement in synaptic plasticity in other brain structures (Balschun and Reymann, 1994; Yasui and Kawasaki, 1995; Crosby et al., 2018; Chen et al., 2019), we then asked if CCK also played a role in corticostriatal STDP. We applied a post–pre pairing STDP protocol where an MSN action potential is evoked a few milliseconds before a cortical stimulation, 100 times at 1 Hz, while monitoring the patch stability via Ri measurements (Fig. 5*A*). As previously described, in control conditions, the post–pre pairing induced a significant LTP (mean EPSC = 137.3% of the baseline; *p* = 0.0026), whereas after bath application of 1 µM LY, post–pre pairing induced a significant LTD (mean EPSC = 82.71% of the baseline; *p* = 0.0190; Fig. 5*B*,*C*). The reversal in the direction of synaptic plasticity during the LY condition was not observed in the presence of the CCK1R antagonist Lorg. Indeed, as in control conditions, a post–pre pairing protocol induced a significant LTP during Lorg condition (mean EPSC = 173.8% of the baseline; *p* = 0.0340; Fig. 5*E*,*F*), demonstrating that CCK2R but not CCK1R induces a switch of plasticity at the S2→MSN synapse.

## Discussion

Despite its abundance and well established modulatory role, the effect of CCK on corticostriatal glutamatergic transmission and plasticity has remained elusive. Using both ex vivo and in vivo electrophysiological approaches, our data show that blocking CCK2R with its specific antagonist, LY225910, modulates synaptic inputs from S2 onto striatal neurons from both the direct and indirect pathways, as identified by optotagging. Moreover, while the CCK1R antagonist Lorg only weakly shortens S2→MSN synaptic events and decreases spike threshold, blocking CCK2R with LY in acute slices significantly decreases the excitability of striatal neurons, shortens and weakens S2→MSN EPSCs, and reverses STDP direction, shifting it from potentiation to depression.

### Integrating multiscale techniques, diverse models, and pharmacological tools to address CCK's modulatory role in MSNs

A key feature of the striatal circuit is the distinction between the direct and indirect pathways (Albin et al., 1989). Therefore, it is essential to understand whether CCK, which is abundant throughout the striatum, similarly affects MSNs in both pathways. Using the D2-ChR2 transgenic mouse line, which effectively discriminates MSN D1 and MSN D2 cells with a combined optical–electrophysiological in vivo approach (Ketzef et al., 2017; Alegre-Cortés et al., 2021), we were able to demonstrate the modulatory role of CCK in S2→MSN synaptic transmission in both direct and indirect basal ganglia pathways. We observed that MSNs in both the direct and indirect pathways responded similarly to CCK. However, we hope that future work could replicate these results on a bigger sample size, thus validating the findings observed here. Once we established that MSNs in both the direct and indirect pathways responded to CCK and given the similarities in circuit anatomy (Hintiryan et al., 2016) and the reported involvement of CCK in synaptic transmission and plasticity, we opted for the traditional ex vivo acute slice preparation over optotagging to allow for a more detailed analysis of intrinsic and synaptic properties (Deng et al., 2010; Crosby et al., 2015, 2018). In addition to leveraging the wealth of knowledge on rat MSN physiology and S2→MSN synaptic physiology (Fino et al., 2005, 2008; Fino and Venance, 2010; Paille et al., 2013), we also took advantage of the extensive literature on CCK receptor expression in the striatum (Van Dijk et al., 1984; Pélaprat et al., 1987) and on the validated pharmacological agents used to modulate and interrogate the CCK circuit. Several selective CCK2R antagonists are available and have been used in literature, including L-740093, L-365260, or LY288513 (Blacker et al., 1997). We chose to use LY225910, as it has become the most commonly used antagonist in recent years, both for application on acute slices (Crosby et al., 2018) and for IP injection (Ballaz et al., 2020; Bernard et al., 2021). Nevertheless, while the use of LY225910 as a CCK2R antagonist is efficient and well established for these approaches, future work should aim at reproducing these results using an alternative CCK2R antagonist to independently confirm that the observed effects are specifically due to the blockade of CCK2R. Finally, while our results highlight the major role of the CCK2R, they also suggest a lesser involvement of the CCK1 receptor in synaptic transmission and excitability of MSNs. Future work on the role of CCK in corticostriatal transmission should therefore take these results into account.

### CCK: where does it come from, and where does it act?

CCK, the most abundant neuropeptide in the brain, is particularly enriched in the striatum. The three primary central sources of striatal CCK identified so far are the midbrain, the nucleus tractus solitarius (NTS), and the cortex, with the cortex being the major source (Hökfelt et al., 2002). In our acute slice model, the horizontal slicing preserved the corticostriatal pathway while excluding the midbrain and NTS pathways, suggesting that CCK may reach the striatum primarily via cortical projections. This is supported by findings that few CCK+ cells have been identified within the striatum itself and primarily in the ventral region which we did not target for patch clamp (Takagi et al., 1984; Hökfelt et al., 1988). However, we cannot exclude the possibility that CCK reaches the striatum through alternative pathways—and a further caveat regarding the in vivo experiments is the effect of anesthesia on CCK levels, as anesthesia may influence CCK release and basal levels (Müller et al., 2011). Our experimental approach does not definitively pinpoint the precise site of CCK action in modulating transmission and plasticity. Given the receptor localization (Pélaprat et al., 1987) and the observed effects of CCK on both MSN intrinsic membrane properties and synaptic responses, the most likely site of action appears to be on MSN dendrites expressing CCK2R. Future studies—both physiological (e.g., paired-pulse stimulation, direct application of exogenous CCK via Picospritzer) and molecular (e.g., examining CCK receptor expression on S2 axon terminals in the striatum)—are needed to clarify these mechanisms.

### CCK modulates synaptic transmission and synaptic plasticity in multiple brain areas

To our knowledge, this is the first study demonstrating CCK's role in modulating both transmission and plasticity at the MSN corticostriatal synapse. These findings align with what has been reported in other brain areas. For example, similar to the changes in glutamatergic transmission we observe between the cortex and MSN with CCK2R antagonism, CCK enhances glutamate release in the hippocampus via presynaptic CCK2R binding (Deng et al., 2010; Xiao et al., 2012; Lau et al., 2022) and facilitates LTP induction with high-frequency stimulation (Chen et al., 2019). Similarly, in the hypothalamus, applying a CCK2R antagonist reverses plasticity, leading to LTP rather than LTD after high-frequency stimulation (Crosby et al., 2018). These findings suggest that CCK binding at CCK2R is essential for the plasticity process across multiple brain structures, including the corticostriatal synapse as shown here. Further studies are needed to elucidate the molecular pathways linking CCK binding CCK2R to the establishment of plasticity and to assess whether these mechanisms are conserved across brain regions. In the hippocampus, CCK's role in plasticity directly involves presynaptic CCK release (Chen et al., 2019; Lau et al., 2022), while in the hypothalamus, it requires the intermediate activation of CCK2R expressed on astrocytes surrounding the synapse (Crosby et al., 2018). However, in the corticostriatal synapse, astrocytic involvement is unlikely, as astrocytes in the DLS do not appear to express CCK2R (Saunders et al., 2018). One hypothesis is that GABA_A_ receptors may be involved, as their inhibition can shift corticostriatal plasticity induced by STDP from LTP to LTD, as observed in our previous study (Paille et al., 2013).

### Behavioral implications of CCK modulation at the corticostriatal synapse

Our work opens up several avenues for further exploration, particularly regarding CCK's broader role in the basal ganglia circuit. Given the complex connectivity and multiple actors involved in this network (Cui et al., 2015; Haber, 2016), CCK appears to be a key modulator. Previous studies have shown that adding CCK to the striatum can enhance dopaminergic and GABAergic transmission by increasing both dopamine and GABA release (Ferraro et al., 1996). Further investigation into CCK's involvement in transmission mechanisms within the striatum could thus reveal its impact on the basal ganglia circuitry as a whole, providing a deeper understanding of its modulatory effects on this critical brain system.

Moreover, since we show that CCK2R activation is necessary to induce LTP, it would be interesting to examine whether blocking CCK2R affects motor learning, particularly for tasks involving basal ganglia activity. While some studies have explored how CCK modulates behavior, most have focused on its effects on stress (Harro and Vasar, 1991; Kõks et al., 2001; Raud et al., 2003; Abramov et al., 2004; Lo et al., 2008; Ballaz et al., 2020), with few investigating its role in motor behavior (Vasar et al., 1991; Blacker et al., 1997; Daugé et al., 2001; Kõks et al., 2001). Some studies have suggested the role of CCKergic signaling in memory consolidation (Fekete et al., 1984; Flood et al., 1995; Huston et al., 1998; Lo et al., 2008; Feng et al., 2021), but most of these have focused on hippocampal function rather than synaptic plasticity in specific structures. The effects of CCK on striatal-dependent motor performance and learning, and the underlying cellular and molecular mechanisms, remain unclear. One complication is that motor behavior is complex, arising from the combined actions of multiple brain areas, including the basal ganglia, motor cortex, cerebellum, and brainstem nuclei. Designing a behavioral task driven exclusively by basal ganglia circuitry could provide insight into whether CCK modulation of corticostriatal transmission impacts behavior.

## Conclusions

For some time, neuropeptides have primarily been used for their convenience in cell tagging. Despite being well known for over 50 years due to their high concentration levels and the presence of their receptors in key brain structures, their powerful role in neuromodulation has often been overlooked. Recent studies, including our own, increasingly highlight the crucial role of CCK in modulating transmission and plasticity across different brain structures (Crosby et al., 2018; Chen et al., 2019; Lau et al., 2022; Martinez Damonte et al., 2023). In this paper, our results showed that CCK is involved in MSN excitability as well as corticostriatal transmission. Interestingly, we also have shown that blocking CCK could completely reverse the LTP induced with an STDP protocol. These findings underscore the importance of considering neuromodulation through neuropeptides as a potent mechanism in neuroscience research.

## Synthesis

Reviewing Editor: Masha Prager-Khoutorsky, McGill University Decisions are customarily a result of the Reviewing Editor and the peer reviewers coming together and discussing their recommendations until a consensus is reached. When revisions are invited, a fact-based synthesis statement explaining their decision and outlining what is needed to prepare a revision will be listed below. The following reviewer(s) agreed to reveal their identity: Christopher Pittenger.

Overall the reviewers acknowledged that many of their comments and suggestions were implicated and the manuscript is significantly improved. However, some additional changes should be introduced mostly to improve the readability of the text.
